# Mutation screening of *HSF4* in 150 age-related cataract patients

**Published:** 2008-10-20

**Authors:** Yuefeng Shi, Xiaohe Shi, Yiping Jin, Aizhu Miao, Lei Bu, Jianyong He, Haisong Jiang, Yi Lu, Xiangyin Kong, Landian Hu

**Affiliations:** 1 Institute of Health Science, Shanghai Institutes for Biological Sciences of CAS and Ruijin Hospital, Shanghai JiaoTong University School of Medicine, Shanghai, China; 2 Shenyang Pharmaceutical University, Shenyang, China; 3 Dongfang Hospital, Shanghai, China; 4 Eye and ENT hospital of Fudan Unversity, Shanghai, China; 5 State Key Laboratory of Medical Genomics, Ruijin Hospital, Shanghai Jiao Tong University School of Medicine, Shanghai, China

## Abstract

**Purpose:**

Heat shock transcription factor 4 (HSF4) regulates the expression of several heat shock protein (HSP) genes. HSPs are one of the major components responsible for lens protein organization. Recently, we found that mutations of *HSF4* result in hereditary cataract. In this study, we explore the role of *HSF4* in the development of age-related cataract.

**Methods:**

We screened sequence variants of *HSF4* in age-related cataract patients and the natural population from Shanghai, China.

**Results:**

In individuals of natural populations, we detected no single nucleotide polymorphism (SNP) with a frequency higher than 5% in a complete coding region or in their exon–intron boundaries. In 150 age-related cataract patients, we identified seven sequence changes. We found an intronic G→A transition (c.1020–25G>A) in one patient, a missense mutation (c.1078A>G) in exon 4 in two patients, a silent mutation (c.1223 C>T) in exon 5 in two patients, an intronic C→T transition (c.1256+25C>T) in one patient, and a silent mutation in exon 6 (c.1286 C>T) in one patient. These five variants were not represented in 220 control individuals. We also identified an intronic C→T transition (c.1019+9C>T) and a missense mutation (c.1243G>A) in exon 3 in three patients, but these two variants were also present in 100 control subjects.

**Conclusions:**

We identified five new *HSF4* mutations in 150 age-related cataract patients, enlarging the spectrum of *HSF4* mutations in cataract patients. This result indicates that *HSF4* mutations account for only a small fraction of age-related cataracts.

## Introduction

Age-related cataract is the leading cause of blindness in the world today [1]. It is a multifactorial disease caused by interactions between gene and environmental factors. Research in age-related cataract has focused mainly on environmental factors, which seem to be important risk factors. However, a recent twin study demonstrated that genetic factors are most important among several factors in age-related cataract [2]. Because age-related cataract is a complex trait, multiple loci are probably involved. Although evidence of a genetic effect on the development and progression of lens opacity is increasing, genes that are clearly associated with adult-onset cataract are few compared with those for hereditary cataract. Heat shock transcription factor 4 (HSF4) is a member of the family of HSFs that mediate the inducible transcription response. HSF4 regulates the genes for heat shock protein 70 (HSP70), HSP90, HSP27, and lens structural protein, αB-crystallin [3,4]. Protein native organization is essential for lens transparency, and HSPs play an important role in maintenance of the supramolecular organization of the lens protein [5]. In 2002, we found that mutations of *HSF4* led to cataract formation [6]. After that, several new mutations were detected in human, mouse, and dog cataracts [7-11]. Moreover, Bagchi et al. [5] revealed a decrease in HSP levels with age and posited that the decrease of HSP might be responsible for the loss of optimal protein organization and eventual appearance of age-related cataract. Certain sequence changes of *HSF4* will permit abnormal expression of HSPs and thereby influence the function or level of HSPs, which might increase the susceptibility to age-related cataract. Therefore, we suspect that HSF4 is a genetic factor associated with age-related cataractogenesis.

## Methods

The study had the approval of the local and regional ethics committees and conformed to the tenets of the Declaration of Helsinki. We based our analysis on 150 sporadic, age-related, cortical cataract patients in Shanghai who were free from diabetes mellitus, high myopia, glaucoma, uveitis, and ocular injury. The degrees of cataract in all eyes were CII or CIII according to the LOCS II system. The degree of nuclear hardness in all eyes were equal to or more than grade III according to the Emery and Little nuclear hardness classification. All the patients then received phacoemulsification and intraocular lens implantation surgery. All patients received careful examination preoperatively and postoperatively including tests of visual acuity and slit-lamp and fundus examinations with the dilated pupil. The unrelated control subjects were collected from Shanghai, China after slit-lamp examination. Patients’ ages ranged from 39 years to 90 years (67.2±13.0 years), controls ranged from 21 years to 61 years (40.4±12.46 years). After obtaining written informed consent, we obtained 5 ml of peripheral blood from each patient for sequence analysis. Genomic DNA was extracted from peripheral blood that we obtained using a Qiagen blood kit (QIAGEN Gmbh, Hilden, Germany).

We designed nine pairs of human *HSF4*-specific oligonucleotide primers to amplify each coding exon and it’s flanking intronic sequences. Using polymerase chain reaction (PCR), we amplified products from genomic DNA and then sequenced the PCR product from both directions with dye terminator methods using an ABI-3100 sequencer (Applied Biosystems, Foster City, CA).

## Results and Discussion

To detect *HSF4* sequence abnormalities, we screened all coding exons and the intron–exon boundaries of *HSF4* (Table 1) by direct genomic PCR sequencing. To identify common variants in *HSF4*, we sequenced 100 unrelated control individuals but found no single nucleotide polymorphism (SNP) with a frequency over 5% in all 13 exons and flanking regions. In seven cases of 150 patients, we found five sequence changes that were not present in 220 control subjects (Table 2). These findings included one missense mutation in exon 4 (c.1078A>G), two silent mutations in exons 5 (c.1223C>T) and 6 (c.1286C>T), two intronic mutations in intron 3 (c.1020–25G>A), and an intronic C→T transition in exon 5 (c.1256+25C>T). We also identified a c.1019+9C>T substitution located 9 bp downstream of the 3′ end of exon 3 and a G→A missense mutation (c.1243G>A) in exon 5 that led to an Arg116>His substitution. These two sequence changes were also present in 100 control subjects (Table 2).

**Table 1:** Primer sequences for mutation screening.

**Primer name**	**Forward primer sequence (5′→3′)**	**Reverse primer sequence (5′→ 3′)**
Exon 3	GCACTTTCCGCGGCTTTGAC	GCAGGCTCCTAACCCTTCTTCG
Exon 4	AGCGCAGGACTGGCCGTGAG	GGGACTGGGTCGCAGGAGCA
Exons 5–6	AGTGCTGCCCCAGTATTTCAAG	GCCAGTTATGGTCTCATCCCG
Exons 7–8	CCCAGCCTCGCCATTCTGTG	TTCCCGGTGAAGGAGTTTCCA
Exons 9–10	AGCTCTGCTGACTTGGCTGC	CACTGACTTCTCCCTCTACCCC
Exon 11	CCAGATGGCTGTAGGGGTAGA	TATCATGGAGTCAAATGGCTAGG
Exon 12	CCTATCATTTTCTAAAGATTGGG	TATGGACCAGAGGGCTTGAC
Exons 13–14	GCCTCTAGATGTGAGTACCCCTT	CCCTGCAAATTGCAGATTGC
Exon 15	AAGGGAGCTAGGCACCGGATC	AGCAGAAGGCAGGCGGGCAG

**Table 2:** *HSF4* mutations in 150 sporadic age related cataract patients.

**Sequence variants**	**Amino acid changes**	**Case/patient**	**Age (gender)/patient**
		**Case/control**	**Age (gender)/control**
c.1019+9C>T	None	3/150	78 (M), 52 (F), 68 (F)
Heterozygous		3/100	40 (F), 35 (F), 44 (M)
c.1020–25G>A	None	1/150	62 (F)
Heterozygous		0/220	
c.1078A>G	Gln→Arg	2/150	77 (F), 56 (M)
Heterozygous		0/220	
c.1223C>T	None	2/150	48 (F), 62 (M)
Heterozygous		0/220	
c.1243G>A	Arg→His	3/150	69 (F), 60 (F), 59 (F)
Heterozygous		2/100	46 (M), 40 (F)
c.1256+25C>T	None	1/150	70 (M)
Heterozygous		0/220	
c.1286C>T	None	1/150	55 (F)
Heterozygous		0/220	

Note. M: Male, F: Female. Substitutions c.1020–25G>A, c.1078A>G, c.1223C>T, c.1256+25C>T and c.1286C>T were not represented in 220 control individuals, substitutions c.1019+9C>T and c.1243G>A were also presented in control subjects.

**Figure 1:**
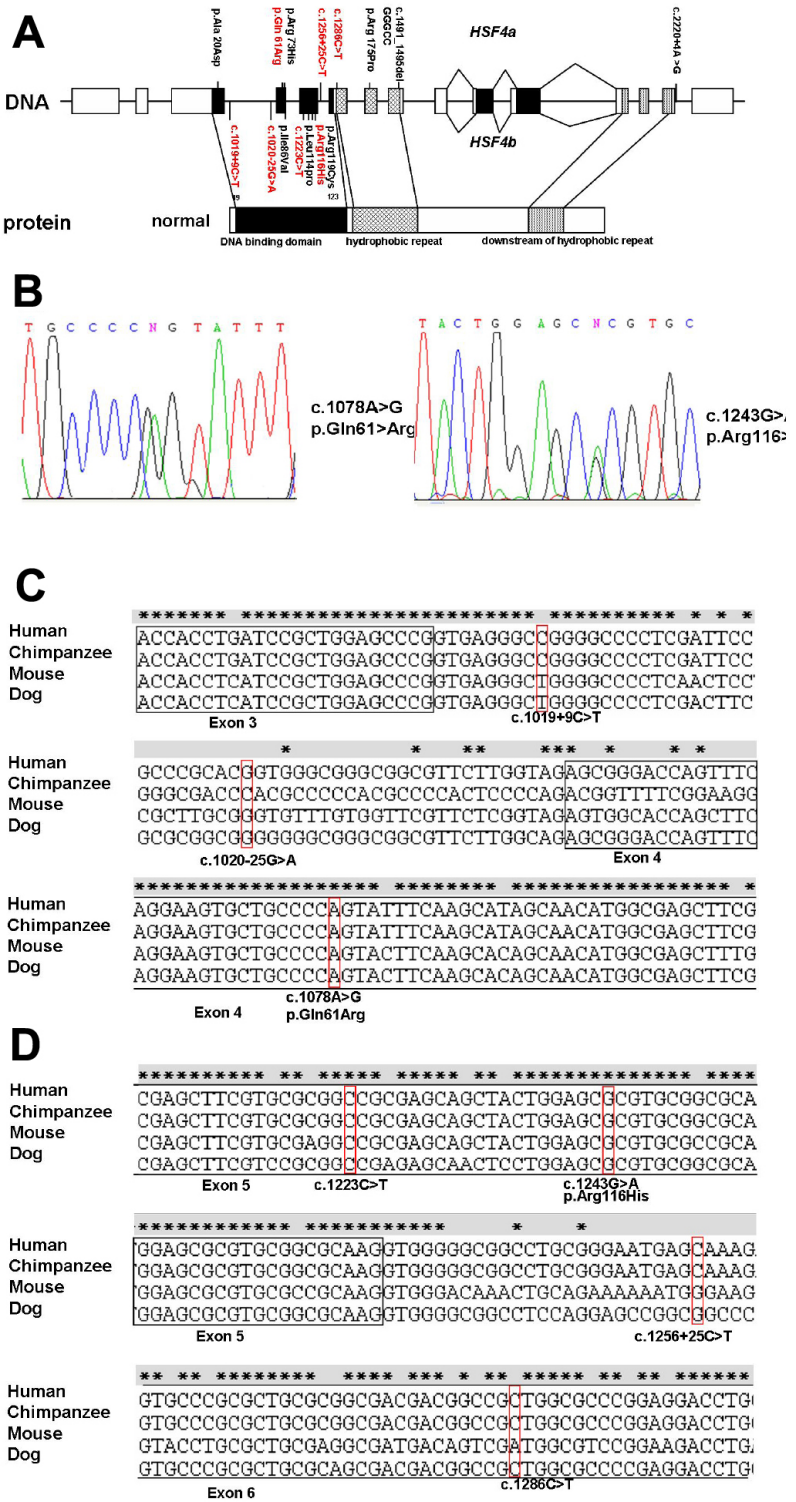
Distribution of variants in *HSF4*. Mutations reported in this study are highlighted in red; previously reported mutations are highlighted in black. **A**: *HSF4* structure is shown in the diagram with the locations of the mutations labeled. **B**: Genomic DNA sequence electropherograms of the two new missense mutations are shown. Left panel, sequence electropherograms from an affected patient heterozygous for the c.1078A>G mutation resulting in a p.Gln61>Arg substitution; right panel, sequence electropherograms from an affected patient heterozygous for the c.1243G>A mutation resulting in a p.Arg116>His substitution. **C**: *HSF4* sequence alignment and reported mutations in this study are given and highlighted.

**Figure 2:**
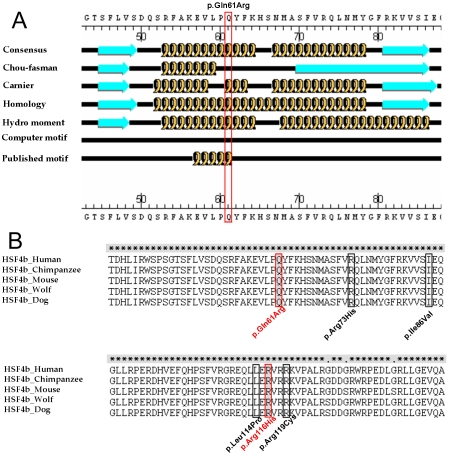
Conserved Gln61 changed to Arg was predicted to disturb an α-helix. p.Gln61>Arg substitution changed one conserved Gln residue and was predicted to disturb an α-helix. **A**: Secondary structure of HSF4b predicted using PepTool Lite software (Biotools Inc., Edmonton, Alberta, Canada). **B**: Multiple sequence alignments of HSF4 homologous sequences in different species are shown. Mutations reported in this study are highlighted in red; previously reported mutations are highlighted in black.

Consistent with the reported *HSF4* mutations in cataract patients (Figure 1), the five patient-specific mutations and the two rare changes reported in this study are clustered in the genomic region that codes for the DNA binding domain (DBD) of the HSF4 protein (Figure 1 and Figure 2). The DBD is the most conserved functional domain of HSF4 in mammals (Figure 2B). Both new missense substitutions identified here are located at this region (Figure 2B). The c.1078A>G missense mutation leads to a p.Gln61>Arg substitution, turning a polar neutral amino acid (Gln) into a basic one (Arg). The p.Gln61>Arg substitution occurred within a predicted α-helix of HSF4 (Figure 2A). Therefore, this mutation might interrupt the α-helix structure of the DBD of HSF4 (Figure 2A). Consistent with our finding, a similar Arg to Gln substitution within the eighth α-helix of the A domain of R-type pyruvate kinase has been previously reported to be associated with hereditary hemolytic anemia [12]. Another missense substitution occurs at a highly conserved Arg residue (p.Arg116>His). As shown in Figure 2B, this residue is flanked by two known disease-causing missense mutations (Figure 2B).Despite the presence of the p.Arg116>His substitution in two controls, we could not exclude the disease-causing role of this missense substitution because the control subjects with p.Arg116>His substitution are only 40 and 46 years old (Table 2) and are likely to suffer age-related cataracts in the future.

Silent mutations could interfere with signals for RNA splicing, RNA folding, microRNA binding, regulation, translation rate, and even protein folding [13-15]. Thus, synonymous changes may contribute to the development of human diseases [16,17]. In 150 age-related cataracts, we found two silent mutations (c.1223C>T and c.1286C>T) and two intronic mutations (c.1020–25G>A and c.1256+25C>T). The c.1020–25G>A substitution just happened to be within the pyrimidine-rich region of the 3′ splicing acceptor of intron 3. Previous studies have suggested that mutations within the pyrimidine-rich intronic sequence can cause human diseases [18-20]. However, because of lack of eye tissues, we could not examine the effect of these four mutations on RNA splicing.

*HSF4* is expressed in a tissue-specific manner [3] and has at least two splice forms, *HSF4a* and *HSF4b* [21]. In the mouse eye, expression of Hsf4 primarily in the form of Hsf4b, which stimulates constitutive and inducible transcription of heat-shock genes, is high [6]. Through binding between heat shock elements and the DBD, the most important functional region of HSF4, HSF4 performs its modulatory function. The seven variants we reported here are located in the region from intron 3 to exon 6 (Figure 1). Exons 3–5 and part of exon 6 encode part of the DNA-binding region of HSF4. This NH_2_-terminal helix–turn–helix DBD is the most conserved functional domain of HSFs. Functional analysis of these seven variants on gene splicing and DNA binding activity of HSF4 is required for understanding the role of these variants on age-related cataract development.

We detected only seven sequence variants in the examined region of *HSF4* in 150 age-related cataract patients. In this regard, *HSF4* coding region mutations account for only a small fraction of age-related cataracts in the Chinese population. Regulatory variations in the promoter region are under investigation.
